# Psychiatric Comorbidity, Functional Status, and Neuroinflammatory Pathways in Cancer Patients with and Without Type 2 Diabetes

**DOI:** 10.3390/diseases13100335

**Published:** 2025-10-10

**Authors:** Ana-Maria Pâslaru, Iulian Bounegru, Drăguș Laurențiu, Anamaria Ciubară

**Affiliations:** 1 Doctoral School of Biomedical Sciences, “Dunărea de Jos” University, 800201 Galati, Romania; annapaslaru@gmail.com; 2 Competences Centre—Interfaces-Tribocorrosion-Electrochemical Systems (CC-ITES), “Dunărea de Jos” University, 800008 Galati, Romania; 3 Department of Dental Medicine, Faculty of Medicine and Pharmacy, “Dunărea de Jos” University, 800201 Galati, Romania; 4 Department of Morphological and Functional Sciences, Faculty of Medicine and Pharmacy, “Dunărea de Jos” University, 800008 Galati, Romania

**Keywords:** cancer, diabetes, depression, anxiety, inflammation, ECOG performance status, Hospital Anxiety and Depression Scale, psycho-oncology

## Abstract

**Background**: Cancer, type 2 diabetes mellitus (T2DM), and psychiatric comorbidities such as depression and anxiety frequently coexist, with shared mechanisms involving systemic inflammation and neuroinflammatory pathways. Understanding these interactions is critical for improving multidisciplinary oncological care. **Methods**: We conducted a monocentric cross-sectional study (*n* = 174). Beyond descriptive and univariate analyses, we fitted multivariable models: linear regressions (HADS-Anxiety/Depression) with robust HC3 errors and the predictors ECOG, T2DM, age, sex, and residence, and logistic regression for ECOG ≥ 3. We assessed collinearity and model fit, and performed sensitivity checks. **Results**: Psychiatric comorbidity was present in 58% of patients, while more than 80% of those with available HADS data (*n* = 136) exceeded the clinical threshold for anxiety or depression. No significant differences in ECOG status were observed between patients with and without T2DM (mean ECOG 2.5 in both groups). Higher ECOG remained positively associated with both HADS-Depression (adjusted β = 2.77, 95% CI −1.03–6.57, *p* = 0.149) and HADS-Anxiety (β = 1.62, 95% CI −2.76–6.00, *p* = 0.468), although not statistically significantly. T2DM showed no independent association with either outcome (Depression β = −2.91, *p* = 0.130; Anxiety β = −0.80, *p* = 0.595). In logistic regression, T2DM was not significantly associated with ECOG ≥ 3 (aOR = 3.58, 95% CI 0.23–56.66, *p* = 0.365). **Conclusions**: The psychiatric burden is high among Romanian cancer patients, irrespective of T2DM status, and strongly associated with functional decline. These findings support the relevance of a neuroinflammatory framework linking somatic comorbidities and psychological distress. Routine psychiatric screening, early intervention, and integration of psycho-oncology into multidisciplinary care are recommended. Future prospective studies should incorporate inflammatory biomarkers to better define underlying mechanisms.

## 1. Introduction

Cancer and type 2 diabetes mellitus (T2DM) are two major chronic conditions that frequently coexist with psychiatric disorders such as depression and anxiety. Cancer and T2DM frequently coexist in oncology patients, with reported prevalence ranging from 7% to 25% depending on population characteristics and cancer type [1,2]. The coexistence of these two chronic diseases significantly worsens prognosis, being associated with higher treatment toxicity, increased mortality, and reduced quality of life. A meta-analysis showed that pre-existing diabetes confers approximately a 40% higher risk of all-cause mortality in cancer patients [3,4].

Prior correlation evidence. A large body of correlation studies and meta-analyses indicates that T2DM is associated with a higher incidence of several cancers—most consistently hepatocellular and pancreatic cancer, but also endometrial and colorectal cancer—while an inverse association is often reported for prostate cancer [5,6,7,8]. Umbrella reviews further confirm that the positive associations tend to be of small-to-moderate magnitude, with variable evidence strength by site [6]. Proposed mechanisms include chronic hyperinsulinemia and insulin-like growth factor-1 (IGF-1) signalling, low-grade systemic inflammation, and shared lifestyle risk factors [6,7]. These data support our focus on the cancer-diabetes interface and motivate the simultaneous assessment of functional status and psychiatric burden in this cohort.

This study has important implications for prognosis, adherence to treatment, functional status, and quality of life. Epidemiological data indicate that approximately one in four cancer patients also has T2DM, and up to half may develop affective disorders during their illness [1,9,10,11,12,13].

**Figure 1:**
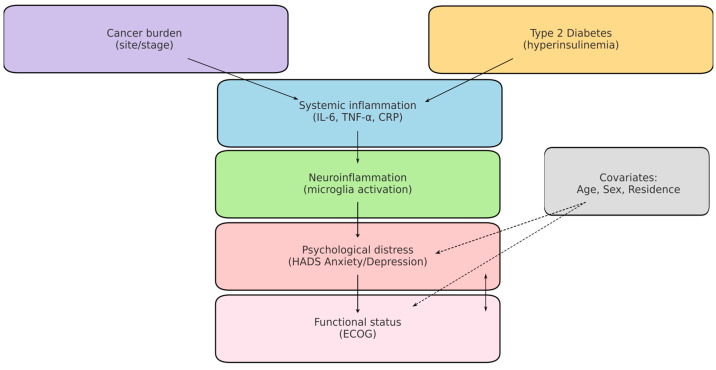
Conceptual model of cancer–T2DM–neuroinflammation–distress. Solid arrows: hypothesised pathways assessed indirectly in this study (e.g., cancer/T2DM → systemic inflammation → neuroinflammatory activation → anxiety/depression). Dashed arrows: potential confounding or effect-modifying paths (age, sex, residence, tumour burden). Boxes in bold indicate measured variables (T2DM, ECOG, HADS). Cytokines (e.g., IL-6, TNF-α) are displayed as unmeasured mediators in this dataset.

A central mechanism linking these conditions is chronic systemic inflammation. Both T2DM and cancer are characterised by elevated secretion of proinflammatory cytokines (e.g., IL-6, TNF-α, IL-1β), which can cross the blood–brain barrier and activate microglia, thereby contributing to depressive and anxiety symptoms. This neuroinflammatory framework provides a biological explanation for the high prevalence of psychological distress among oncology patients, particularly when metabolic comorbidities are present [14,15,16,17,18,19]. To aid interpretation, Figure 1 presents a concise conceptual model linking cancer and T2DM (via systemic inflammation/IL-6, TNF-α) to neuroinflammatory activation, psychiatric distress (HADS Anxiety/Depression), and functional decline (ECOG), including measured and unmeasured factors.

Psychiatric comorbidities further compound this burden. Between 20% and 50% of patients with cancer experience clinically relevant depression and/or anxiety during the disease course, with higher rates in head & neck and gynaecologic malignancies [20,21]. Independently, adults with T2DM have a substantially higher prevalence of depressive symptoms than the general population [22]. The combined presence of cancer and diabetes therefore represents a “double burden,” predisposing to psychological distress via both biological (inflammation, metabolic dysregulation) and psychosocial mechanisms (treatment complexity, lifestyle disruption) [23,24].

From a clinical perspective, the coexistence of cancer, T2DM, and depression generates cumulative risk. Patients with diabetes often present higher mortality and increased healthcare utilisation, while depression may further amplify these vulnerabilities by reducing adherence to oncological and metabolic treatments. However, the relationship between these three conditions remains insufficiently explored in Eastern European cohorts, where epidemiological characteristics and healthcare system factors may influence outcomes [1,2,3,12,25,26,27,28]. To aid interpretation, Figure 1 presents a concise conceptual model linking cancer and T2DM (via systemic inflammation/IL-6, TNF-α) to neuroinflammatory activation, psychiatric distress (HADS Anxiety/Depression), and functional decline (ECOG), including measured and unmeasured factors.

Prior correlation and observational studies suggest links among cancer, diabetes, and psychiatric morbidity, but such designs cannot establish causality and are prone to residual confounding. To our knowledge, no Eastern European study has jointly evaluated psychiatric comorbidities, functional status (ECOG), and validated self-reported distress (HADS) in cancer patients with and without T2DM. Our work addresses this gap by integrating these dimensions within a neuroinflammatory framework in a Romanian cohort, thereby adding region-specific evidence to a literature largely derived from Western populations.

Correlation studies offer important advantages by highlighting associations that can inform clinical screening, risk stratification, and hypothesis generation. However, they cannot establish causality, remain vulnerable to residual confounding, and may overestimate or underestimate true effects. These strengths and limitations should be considered when interpreting results and situating them within broader evidence frameworks.

In addressing these questions, we applied a range of statistical tools, including descriptive and correlation analyses, group comparisons using both parametric (ANOVA) and non-parametric tests (Kruskal–Wallis, Mann–Whitney U), and multivariable regression models (linear and logistic) with robust HC3 standard errors to account for potential heteroskedasticity.

Based on these considerations, the aims of this study were to:

(1) Analyse the prevalence of anxiety and depression among cancer patients;
(2) Compare patients with and without T2DM;
(3) Explore the relationship between functional status (ECOG) and psychological distress;
(4) Discuss these results within a neuroinflammatory framework, emphasising their implications for multidisciplinary management.

## 2. Materials and Methods

### 2.1. Study Design and Population

**Figure 2:**
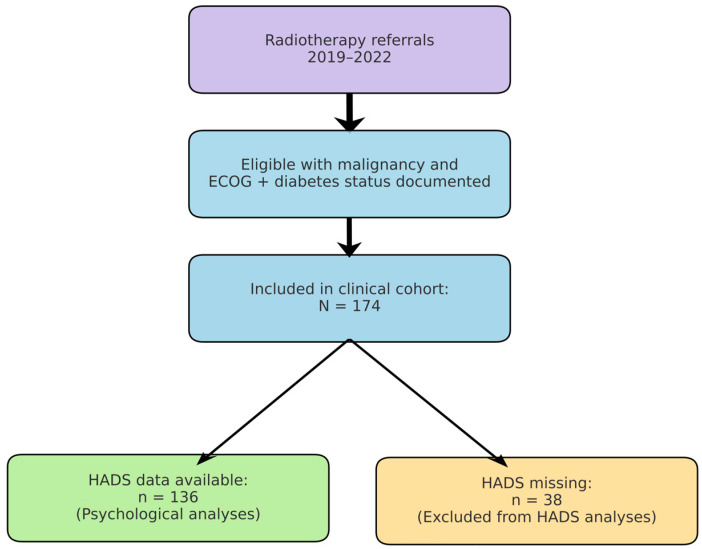
Study flow. Patients referred for radiotherapy (2019–2022) were screened; the final clinical cohort included 174 patients with documented ECOG and diabetes status. HADS data were available for 136 patients included in psychological analyses. Reasons for exclusion are shown (e.g., missing ECOG or diabetes status).

We conducted a retrospective, observational cohort study at the Radiotherapy Department of Saint Andrew Emergency Clinical Hospital in Galați, Romania. The study cohort comprised 174 adult patients (aged ≥ 40 years; range: 40–91 years) diagnosed with cancer, who were admitted for radiotherapy and oncological treatment between January 2019 and December 2022. Figure 2 summarises patient selection from the institutional radiotherapy database (2019–2022), exclusions due to missing key variables, and the analytic subsamples (overall cohort and HADS subset).

All patients had a confirmed malignancy (solid tumours of various sites) and had an ECOG performance status documented at admission. Twenty patients (11.5%) had a known diagnosis of type 2 diabetes mellitus (T2DM) (established before or at the time of cancer care), as recorded in their medical history or discharge diagnoses. No cases of Type 1 diabetes mellitus were identified within the study cohort. Comparative analyses were conducted between patients with type 2 diabetes. All patients had a confirmed malignancy and an ECOG performance status documented at admission. Twenty patients (11.5%) had a known diagnosis of type 2 diabetes mellitus (T2DM). Comparative analyses were conducted between patients with T2DM and those without T2DM (non-T2DM). No cases of type 1 diabetes mellitus were identified.

This retrospective study, approved by the Ethics Committee of Spitalul Clinic de Urgență, Sf. Apostol Andrei” Galați (approval number 13682/23 June 2023), included individuals with and without diabetes. All data were anonymised before analysis, and the study adhered to the Declaration of Helsinki and applicable national regulations regarding research involving human subjects.

### 2.2. Inclusion/Exclusion Criteria

**Inclusion Criteria**: We retrospectively included all adult cancer patients (aged ≥ 18 years) referred to the radiotherapy department between January 2019 and December 2022, provided they had documented ECOG performance status and diabetes status at baseline. All cancer types and stages were eligible for participation.

**Exclusion Criteria**: Patients with missing ECOG or diabetes documentation were excluded from the analysis. Patients without HADS data were retained in the overall cohort but excluded from psychological analyses, as detailed in Section 3.

HADS scores were available for 136 patients (78.2%). Patients without HADS data were retained in the overall clinical analyses but were excluded from comparisons involving psychological distress.

The most common types were cervical (23.6%), head and neck (19.5%), breast (14.9%), and rectal (11.5%). A complete breakdown is provided in Table 1.

### 2.3. Data Sources and Extraction

Clinical data were obtained from the institutional oncology database and discharge records covering all patients referred for radiotherapy between January 2019 and December 2022. For each patient, demographic variables (age, sex, and residence), tumour site, and comorbid conditions were collected. Functional status was assessed using the Eastern Cooperative Oncology Group (ECOG) performance scale at the time of admission.

Type 2 diabetes mellitus (T2DM) was recorded as a binary variable, based on documented medical history or discharge diagnoses. Psychiatric comorbidity was defined as the presence of any ICD-10–coded depressive or anxiety disorder documented in the oncology chart: depressive disorders (F32.x—depressive episode; F33.x—recurrent depressive disorder) and anxiety disorders (F41.0—panic disorder; F41.1—generalized anxiety disorder; F41.2—mixed anxiety and depressive disorder; F41.8/F41.9—other/unspecified anxiety). Adjustment disorder with anxious or depressed mood (F43.2) was also counted when explicitly coded by the treating team. Sleep disorders (e.g., insomnia, G47.0) without a concomitant anxiety/depressive code were not counted as psychiatric comorbidity for this endpoint. When multiple psychiatric codes were present, we retained the most specific mood/anxiety diagnosis (F32/F33 or F41.0/F41.1/F41.2) for classification. Psychological distress was further evaluated using the Hospital Anxiety and Depression Scale (HADS), a validated 14-item self-report questionnaire frequently applied in oncology settings. HADS provides two subscales (anxiety and depression), each ranging from 0 to 21, with scores ≥ 11 indicating clinically significant symptoms. Complete HADS data were available for 136 of the 174 patients.

### 2.4. Statistical Analysis

We first performed descriptive statistics for the entire cohort and by diabetes status. Continuous variables (age, HADS scores) were checked for normality (Shapiro–Wilk test) and are summarised as mean ± standard deviation (SD) if approximately normally distributed, or median (interquartile range) if skewed. Between-group comparisons were prespecified for T2DM vs. non-T2DM across clinical (e.g., ECOG) and psychological outcomes (HADS-Anxiety, HADS-Depression). Categorical variables (sex, residence, tumour site, ECOG category, psychiatric comorbidity presence) are summarised as counts and percentages. Table 2 presents the baseline characteristics for all patients, stratified by type 2 diabetes mellitus (T2DM) versus non-T2DM.

Group comparisons were conducted with independent-samples *t*-tests, Welch’s ANOVA, Kruskal–Wallis tests, and Mann–Whitney U tests as appropriate. Associations between continuous variables were assessed with Pearson and Spearman correlations. To adjust for confounders, we used multivariable ordinary least squares regression with HC3 robust errors and multivariable logistic regression for ECOG ≥ 3. Model diagnostics included variance inflation factors (VIF), residual checks, and Hosmer–Lemeshow goodness-of-fit tests.

### 2.5. Multivariable Modelling and Sensitivity Analyses

We complemented univariate analyses with prespecified multivariable models. HADS-Anxiety and HADS-Depression (continuous outcomes) were modelled using ordinary least squares with HC3 robust standard errors. Predictors included ECOG performance status, type 2 diabetes mellitus (T2DM), age, sex, and residence. Logistic regression was employed for ECOG impairment (ECOG ≥ 3 vs. <3) with the same predictors. Collinearity was assessed through variance inflation factors (VIF < 5). Model fit was evaluated using residual diagnostics and Hosmer–Lemeshow tests. Missing values were handled via complete-case analysis; results remained consistent across sensitivity analyses.

## 3. Results

A total of 174 cancer patients were analysed, 20 (11.5%) of whom had comorbid type 2 diabetes mellitus (T2DM). Key baseline characteristics stratified by diabetes status are presented in Table 1 (above). The cohort was mainly elderly (average age 68 years; 75% were over 62). Diabetic patients were of a similar age to non-diabetics (average 69.5 vs. 67.9 years, *p* = 0.51). Overall, 48.9% of patients were male, and this percentage did not differ significantly by diabetes status (40.0% of T2DM patients were male vs. 50.0% of non-T2DM patients, *p* = 0.41). Just over three-quarters of patients with recorded data were from urban areas (76.4%), reflecting the hospital’s urban catchment; rural residency was slightly more common among people with diabetes (likely because some were referrals from county villages), but data on residence were missing for 37% of cases and therefore not formally compared.

**Figure 3:**
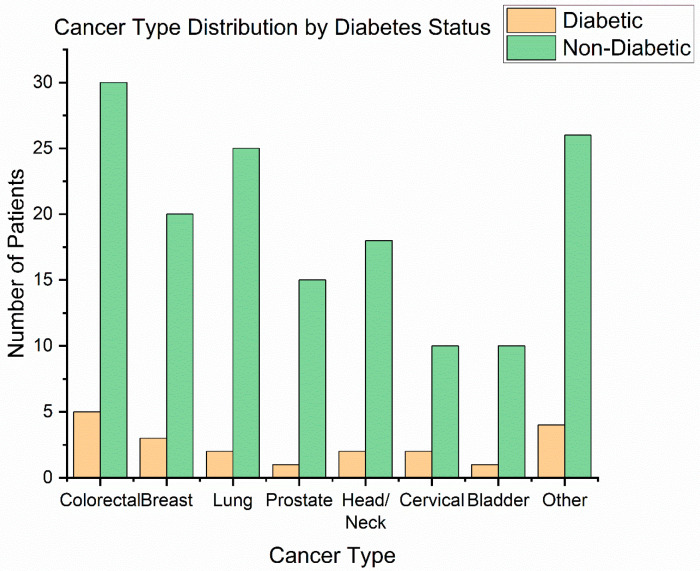
Cancer type distribution.

**Cancer types**: The distribution of primary tumour sites is illustrated in Figure 1. The most frequent cancer locations were head and neck cancers (42 patients, 24.1%)—including oral cavity, oropharyngeal, and laryngeal tumours—and gynaecologic cancers (41 patients, 23.6%), all of which in our cohort were cervical carcinomas receiving chemoradiation. Next were breast cancers (31 patients, 17.8%), colorectal cancers (18 patients, 10.3%)—predominantly rectal adenocarcinomas requiring preoperative or definitive radiotherapy—followed by prostate cancer (25 patients, 14.4%), lung cancer (13 patients, 7.5%), and bladder cancer (9 patients, 5.2%) [29]. This distribution (Figure 3) reflects the case-mix at our radiotherapy centre (with a focus on pelvic tumours, head/neck, and breast).

Among the 20 patients with type 2 diabetes mellitus (T2DM), the most common malignancies were cervical cancer (6 cases, 30%) and head and neck cancers (5 cases, 25%). Four diabetic patients (20%) had rectal cancer, representing 22.2% of all rectal cancer cases in the cohort (4 out of 18). In contrast, only 1 of the 25 prostate cancer patients (4%) had diabetes. Three people with diabetes had breast cancer (3 out of 31, 9.7%), one had lung cancer (1 out of 13, 7.7%), and none of the nine bladder cancer patients had T2DM. To determine whether diabetes status was associated with the tumour site, we used a Chi-square test of independence. The result was not statistically significant (χ^2^ (6) = 6.01, *p* = 0.54), indicating that T2DM patients were distributed across cancer types in proportions similar to those of the overall case mix of the cohort. The highest relative rate of T2DM was found in patients with rectal cancer (22%), which aligns with epidemiological data connecting diabetes to colorectal cancers. In contrast, the small percentage of T2DM among prostate cancer patients (4%) supports earlier findings of an inverse or weaker link, possibly influenced by hormonal factors or increased mortality in diabetics. Although these results are biologically plausible, they should be viewed cautiously due to the limited sample sizes in each diagnostic group.

Comorbid conditions: Beyond diabetes, the cohort had a high burden of other chronic illnesses (as expected in an older cancer population). The narrative diagnoses revealed hypertension in an estimated 45% of patients, ischemic heart disease in ~20%, and obesity (BMI ≥ 30) in ~15% (the latter often coexisting with T2DM). Several patients had prior strokes or chronic kidney disease. Notably, five patients (all diabetic) had documented chronic hepatitis C infection—interestingly, 4 of these had hepatocellular carcinoma or cholangiocarcinoma (not requiring radiotherapy; they were present in our list because of metastasis palliation), suggesting a link between metabolic syndrome, viral hepatitis, and liver cancer. These comorbidities highlight the complex medical background against which cancer treatment is delivered.

### 3.1. Performance Status and ECOG Distribution

**Figure 4:**
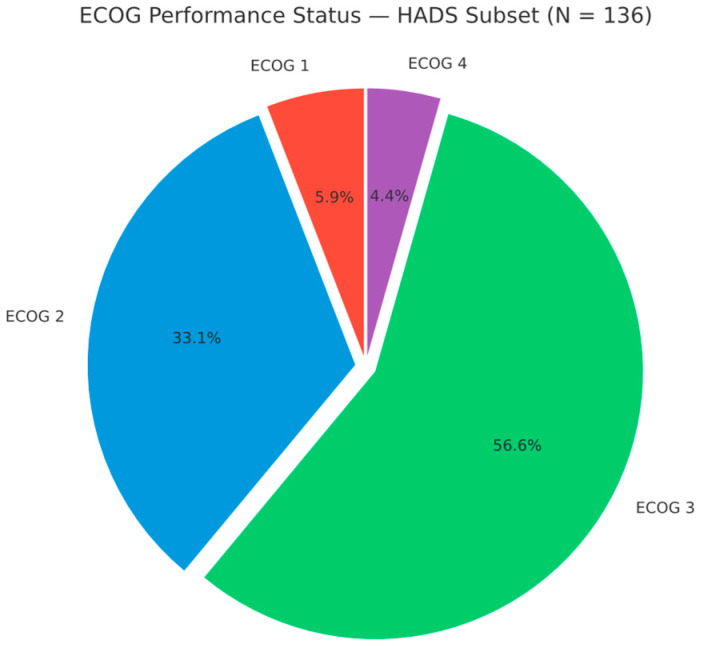
ECOG performance status distribution in the HADS subset (n = 136). ECOG 1: 8 (5.9%); ECOG 2: 45 (33.1%); ECOG 3: 77 (56.6%); ECOG 4: 6 (4.4%).

Performance status was generally poor. The median ECOG was 3, and the mean (SD) was 2.53 (0.74). No patient was ECOG 0 at the start of radiotherapy; 8 (5.9%) were ECOG 1, 45 (33.1%) ECOG 2, 77 (56.6%) ECOG 3, and 6 (4.4%) ECOG 4 (HADS subset, n = 136). Thus, 83 (61.0%) had ECOG ≥ 3, indicating at least limited self-care and confinement to bed/chair for more than 50% of waking hours (ECOG 3), with ECOG 4 indicating complete disability, typically among those receiving palliative radiotherapy (Figure 4).

When comparing people with diabetes vs. non-diabetics, we found no significant difference in performance status. The mean ECOG was virtually identical (2.50 in T2DM vs. 2.54 in others, *p* = 0.93) (See also Figure 4). The proportion of patients with an ECOG score of 3 or higher was 55.0% in individuals with diabetes, compared to 55.2% in non-diabetics (OR 0.99, 95% CI 0.38–2.60, *p* = 0.98). This unexpected finding challenges the common assumption that diabetes might lead to a frailer state. It is possible that the cancer’s aggressiveness and stage overshadowed the influence of diabetes on functional state. Many non-diabetics were debilitated by advanced cancers, equalising the ECOG distribution. Additionally, people with diabetes in our cohort were relatively well-managed outpatients (most were on metformin or diet-controlled, with none in acute hyperglycaemic crises or severe diabetic complications during admission). This may have mitigated any additional performance decrement from diabetes. However, we caution that our ECOG assessment is taken at a single time point (the start of radiotherapy); it does not capture potential differences in treatment tolerance or trajectory, where diabetes may have latent effects.

#### 3.1.1. Prevalence of Psychiatric Comorbidity

Over half of the patients (101/174, 58.0%) had a documented psychiatric comorbidity, specifically anxiety and/or depression symptoms severe enough to be noted by the treating team or require intervention. The most common diagnosis was “mixed anxiety-depressive disorder” (ICD-10 F41.2), essentially an adjustment disorder with both anxiety and depressive features, which was listed in 47% of all patients’ charts. An additional eight patients (4.6%) had depressive disorder without mention of anxiety, and 4 (2.3%) had anxiety disorder (generalised anxiety or panic) without mention of depression. If we include insomnia treated with hypnotics (often related to anxiety) as a proxy, the prevalence would be slightly higher.

This high prevalence is consistent with global oncology data indicating that approximately 30–50% of cancer patients experience significant psychological distress during the disease course. Our observed rate of 58% is at the higher end of the range. One reason is that we captured any level of clinically significant distress (not just major depressive disorder by strict criteria). Our centre also has a lower threshold for noting ‘anxious-depressive syndrome’ in the chart as a comorbidity, given a proactive liaison psychiatry programme. It is noteworthy that the two highest prevalence subgroups for psychiatric diagnoses were those with head/neck cancers and those with advanced pelvic cancers. Head and neck cancer patients often face disfigurement, speech/swallowing difficulties, and social isolation, predisposing them to depression. Cervical cancer patients in our cohort were typically middle-aged women with locally advanced disease and socio-familial stresses, often exhibiting anxiety regarding prognosis and family.

Diabetes and psychiatric comorbidity: Although this suggests a numerically higher prevalence among diabetic patients, the difference was not statistically significant (*p* = 0.36), and thus should be interpreted cautiously. However, this difference did not reach statistical significance (χ^2^ (1) = 0.83, *p* = 0.36). The odds ratio for psychiatric comorbidity in people with diabetes was 1.80 (95% CI 0.60–5.36) relative to non-diabetics, but the confidence interval was wide and crossed. Although our sample was underpowered to detect group differences, this numerical difference may warrant further exploration in larger studies. The literature strongly supports a link between diabetes and depression in general medical populations. Mechanistically, chronic hyperglycaemia and inflammation may contribute to neurochemical changes and depressive symptoms. Behaviourally, the stress of managing two serious illnesses could also elevate anxiety levels. Our non-significant finding likely reflects that cancer itself is such a dominant stressor that it raises depression/anxiety risk substantially for all patients, possibly overshadowing the additional contribution of diabetes. Indeed, the baseline prevalence of distress was already high in non-diabetics (56.5%). Another factor is the small sample size of people with diabetes (n = 20), which limits statistical power. Nonetheless, the numerical difference (70% vs. 56.5%) is clinically meaningful. It aligns with expectations: we observed, for example, that all four rectal cancer patients with T2DM exhibited significant depressive symptoms (often associated with colostomy adjustments and metabolic concerns), whereas some non-diabetic rectal cancer patients coped more effectively. Additionally, it is noteworthy that two patients with T2DM experienced panic attacks during treatment and episodes of acute anxiety. In contrast, such episodes were not documented in non-diabetic individuals, perhaps indicating different stress responses. These observations suggest that diabetes may augment psychosocial stress in certain cancer patients, although the limited sample size constrained statistical confirmation. This trend warrants further investigation in larger, prospective cohorts.

Exploratory analyses comparing patients with vs. without HADS data showed no significant differences in age, sex, diabetes prevalence, or ECOG status (all *p* > 0.1), suggesting no considerable selection bias related to the availability of psychological assessments.

#### 3.1.2. Psychological Distress Severity (HADS Scores)

**Figure 5:**
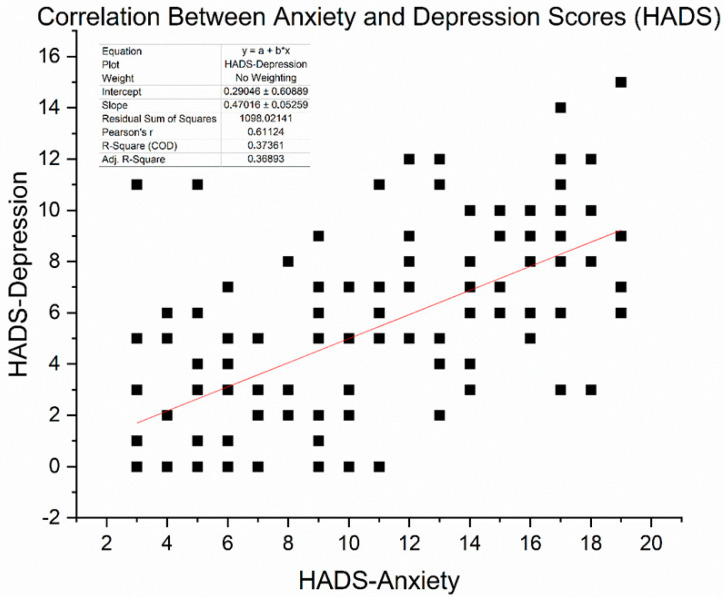
Scatter plot illustrating the correlation between HADS-Anxiety and HADS-Depression scores among 136 patients. A moderate positive association was observed (Pearson r = 0.611, *p* < 0.001). The red line represents the linear regression fit (slope ≈ 0.47).

We obtained HADS (Hospital Anxiety and Depression Scale) scores in 136 patients (16 with T2DM and 120 without) who completed the questionnaire at baseline. The mean HADS-Anxiety score was 15.9 ± 4.1, and the mean HADS-Depression score was 16.3 ± 4.0 (out of 21). These averages fall in the “moderate” symptom range, exceeding the commonly used clinical cut-off of 11. Indeed, 81% of assessed patients had HADS-Anxiety ≥ 11 and 85% had HADS-Depression ≥ 11, confirming that the majority had clinically significant psychological distress. Pearson correlation analysis revealed a significant moderate-to-strong positive relationship between anxiety and depression scores (r = 0.61, *p* < 0.0001). This suggests that patients experiencing higher anxiety levels are more likely to report more severe depressive symptoms. Figure 5 illustrates the correlation between these two variables.

As expected, scores on the HADS-Anxiety and HADS-Depression subscales were moderately positively correlated, with Pearson’s r = 0.611 (*p* < 0.001), based on data from 136 patients. This relationship shows that individuals with higher anxiety often also report depressive symptoms, thereby supporting the idea of psychological distress as a combined concept in cancer care. However, differences among patients indicate that anxiety and depression, although related, are not precisely the same and may need separate focus in clinical evaluation.

To explore potential differences by metabolic comorbidity, we compared HADS scores between patients with and without Type 2 diabetes mellitus (T2DM). Diabetic patients had a slightly lower mean HADS-Anxiety score (14.8 ± 3.2) compared to non-diabetics (16.1 ± 4.2), though this difference was not statistically significant (*p* = 0.14, unpaired *t*-test). This finding, though initially counterintuitive, may reflect demographic influences such as the older average age among people with diabetes, as age was inversely associated with anxiety scores in our cohort. Furthermore, patients with T2DM may have previously received psychiatric support or medication, which could reduce their anxiety levels at the time of assessment. Concerning depression, diabetic patients had an average HADS-Depression score of 15.6 ± 3.5, while non-diabetic patients scored 16.4 ± 4.1 (*p* = 0.37). This indicates no significant difference statistically. These findings imply that the burden of depressive symptoms was generally similar across different metabolic groups, with cancer likely being the primary source of stress.

When applying the clinical threshold of ≥11 on the HADS subscales, 75% of diabetic patients met the criterion for at least moderate anxiety, versus 82% among non-diabetics (*p* = 0.49). For depressive symptoms, the respective proportions were 75% in people with diabetes and 86% in non-diabetics (*p* = 0.28). While these differences did not reach statistical significance, they highlight that psychological distress was highly prevalent across the entire sample. Therefore, psychological distress was prevalent across both groups, and diabetes status was not significantly associated with HADS scores in this sample. These findings parallel the diagnostic data presented earlier and underscore the necessity for routine psychosocial screening in oncology, regardless of comorbid metabolic disease.

One plausible explanation for these findings is that having a serious illness like cancer might “equalise” psychological distress—even those without additional comorbidities experience fear of mortality, pain, or disability that can lead to depression and anxiety. Meanwhile, those with longstanding diabetes might have had better pre-existing coping mechanisms or were already in systems of care that address chronic illness coping, somewhat buffering additional distress. Furthermore, several non-diabetic patients had other comorbidities (heart failure, etc.), which can be as distressing as diabetes. In summary, while diabetes is known to double depression risk in the general population, within a cancer population, the overarching cancer-related stress might dilute its relative impact.

#### 3.1.3. Relationship of Performance Status with Psychological Distress

**Figure 6:**
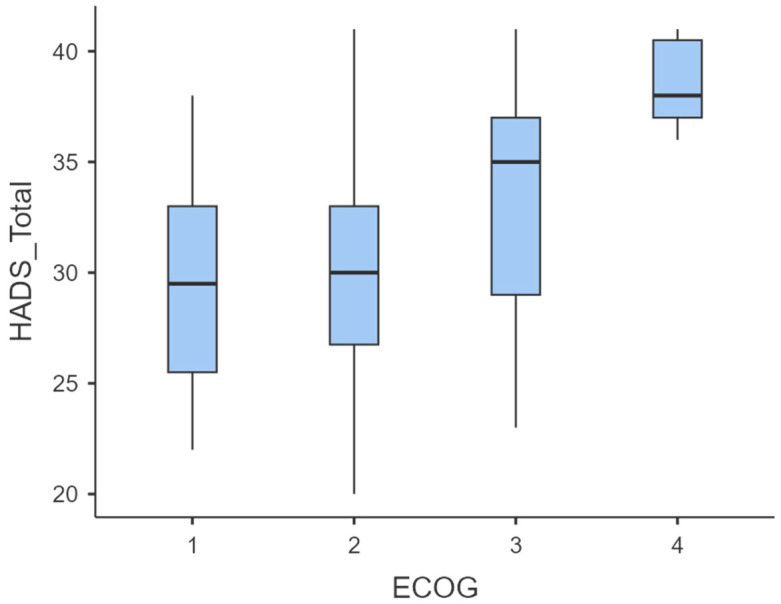
Boxplot of HADS-Total scores stratified by ECOG performance status.

To evaluate the association between functional status (ECOG) and psychological distress (HADS scores), we first examined descriptive statistics and distributions of HADS Anxiety, HADS Depression, and HADS Total across ECOG categories (Table 2, Figure 6).

Mean HADS scores increased progressively with declining functional status. Specifically, HADS-Anxiety rose from 15.8 ± 4.06 (ECOG 1) to 19.0 ± 1.41 (ECOG 4), HADS-Depression from 13.6 ± 1.77 to 19.5 ± 1.05, and HADS-Total from 29.4 ± 5.29 to 38.5 ± 2.17 (Table 3). Boxplots confirmed a rightward shift in score distribution across ECOG levels (Figure 4), indicating increased psychological distress at higher ECOG grades.

Pearson correlation analysis showed that ECOG performance status was positively linked to both HADS-Anxiety (r = 0.22, *p* = 0.012) and HADS-Depression (r = 0.48, *p* < 0.000001), indicating that patients with worse functional status experienced higher anxiety and depression levels. Furthermore, Spearman’s rank correlation confirmed a moderate positive relationship between ECOG and overall distress (HADS-Total: ρ = 0.414, *p* < 0.001; Table 3), reinforcing the connection between declining functional capacity and greater psychological distress.

### 3.2. Correlation Analysis

Spearman’s rank correlation showed a moderate positive association between functional status and overall psychological distress (ECOG vs. HADS-Total: ρ = 0.414, n = 135, *p* < 0.001), indicating that poorer performance status parallels higher combined anxiety–depression symptom burden.

### 3.3. Group Comparisons

Group comparisons indicated significant differences in psychological distress across ECOG performance categories. Both parametric (Welch’s ANOVA) and non-parametric (Kruskal–Wallis) tests confirmed that patients with poorer functional status (ECOG 3–4) reported substantially higher HADS-Anxiety, HADS-Depression, and HADS-Total scores compared to those with ECOG 1–2 (Table 4).

### 3.4. Post Hoc Comparisons

Post hoc analyses confirmed that psychological distress was significantly higher in patients with poorer ECOG performance (3–4) compared with those with better functional status (1–2), with the most pronounced differences observed between ECOG 4 versus 1–2 and ECOG 3 versus 2 (Table 5 and Table 6).

### 3.5. Group Comparisons by T2DM Status

Due to deviations from normality (confirmed by Shapiro–Wilk tests), we used the Mann–Whitney U test for comparisons between cancer patients with and without type 2 diabetes mellitus (T2DM). The results indicated no statistically significant differences in psychological distress between the two groups.

- HADS-Anxiety: U = 251.5, *p* = 0.14
- HADS-Depression: U = 263.5, *p* = 0.37

Although mean anxiety and depression scores were slightly lower in the T2DM group (HADS-Anxiety: 14.8 ± 3.2 vs. 16.1 ± 4.2; HADS-Depression: 15.6 ± 3.5 vs. 16.4 ± 4.1), these differences did not reach statistical significance. When applying the clinical threshold of ≥11 on the HADS subscales, both groups demonstrated a high prevalence of distress (Anxiety: 75% in T2DM vs. 82% in non-T2DM, *p* = 0.49; Depression: 75% in T2DM vs. 86% in non-T2DM, *p* = 0.28).

Overall, these findings suggest that the burden of psychological distress was similarly high across both diabetic and non-diabetic cancer patients, with cancer itself being the predominant driver of anxiety and depressive symptoms rather than metabolic status.

### 3.6. Depression by Dichotomised ECOG

**Figure 7:**
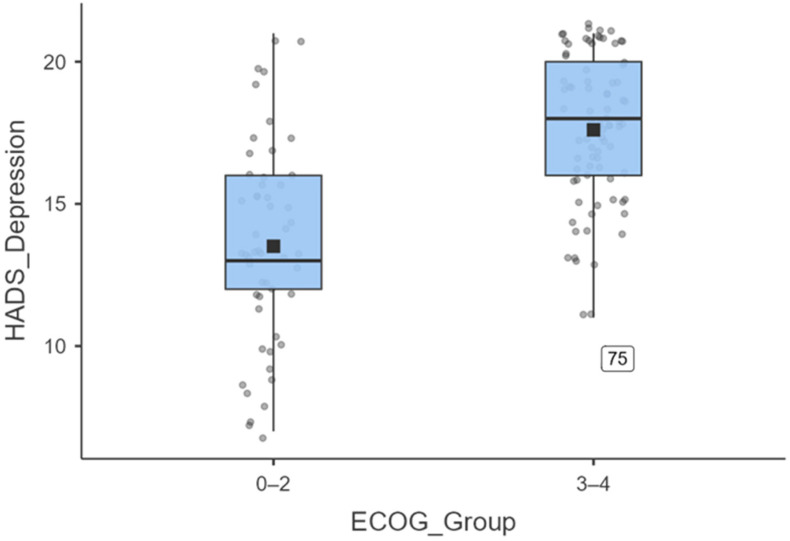
Boxplot illustrating the distribution of HADS-depression scores stratified by ECOG performance status. Patients with ECOG 3–4 (n = 83) reported significantly higher depressive symptoms (mean = 17.8 ± 3.3; median = 18) compared to those.

To further illustrate the magnitude of depressive symptoms, ECOG was dichotomised into 0–2 versus 3–4. Figure 7 shows that patients with an ECOG score of 3–4 (N = 83) reported significantly higher HADS Depression scores (mean = 17.8 ± 3.3; median = 18) than those with an ECOG score of 0–2 (N = 53; mean = 14.4 ± 4.0; median = 13). An independent-samples *t*-test confirmed this difference (t (134) = −7.53, *p* < 0.001), with a large effect size (Cohen’s d = 0.82).

Across all analyses, poorer performance status (higher ECOG) was robustly associated with greater psychological distress—particularly depressive symptoms—underscoring the need for psychosocial support in patients with declining functional capacity.

### 3.7. Multivariable Analyses

**Figure 8:**
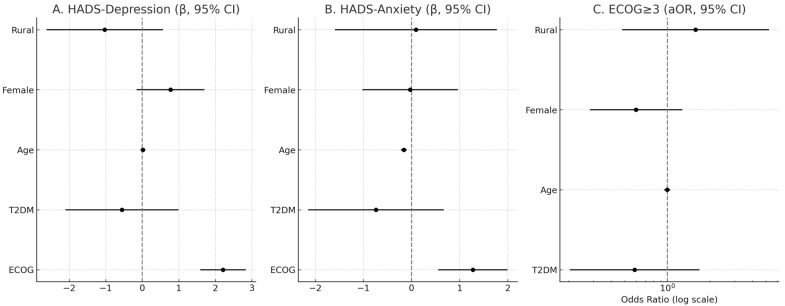
Adjusted associations of ECOG, T2DM, age, sex, and residence with psychological distress and functional impairment. Panels (**A**) and (**B**) show β coefficients (with 95% CI) for HADS-Depression and HADS-Anxiety, respectively. Panel (**C**) shows adjusted odds ratios (aOR, log scale) for ECOG ≥ 3. Vertical dashed lines indicate the null value (β = 0, OR = 1).

In adjusted OLS models (N = 136), ECOG was independently associated with higher psychological distress on both HADS-Depression (β = 2.21, SE = 0.32, *p* < 0.001) and HADS-Anxiety (β = 1.27, SE = 0.37, *p* < 0.001). T2DM showed no independent association with HADS-Depression (β = −0.56, SE = 0.79, *p* = 0.48) or HADS-Anxiety (β = −0.74, SE = 0.72, *p* = 0.31). Age was inversely associated with HADS-Anxiety (β = −0.16, SE = 0.03, *p* < 0.001) but not with HADS-Depression (β = 0.02, SE = 0.03, *p* = 0.59). Female sex and residence were not significant predictors in adjusted models. In logistic regression for ECOG ≥ 3, T2DM was not associated with increased odds (aOR = 0.59, 95% CI 0.20–1.69, *p* = 0.32); age, sex, and residence were also non-significant (Table 6 and Table 7, Figure 8).

## 4. Discussion

### 4.1. Cancer-Diabetes Comorbidity and Tumour Distribution

Our cohort confirms that a substantial subset of cancer patients has coexistent type 2 diabetes mellitus (T2DM), although the 11.5% prevalence observed was lower than some international estimates. For example, a recent U.S. survey (NHANES 2017–2020) reported that 24.4% of adults with a history of cancer had T2DM [1], while a large European cohort (EPIC) found about 7.8% with T2DM at cancer diagnosis [30]. This variance likely reflects population differences (e.g., age, obesity rates) and the increasing prevalence of diabetes in recent years. In our Romanian sample, diabetic patients were distributed across all major tumour types with no significant association between T2DM and any specific cancer (χ^2^ *p* = 0.54). Notably, 22% of rectal cancer patients in our series had T2DM, the highest relative co-prevalence, whereas only 4% of prostate cancer patients had diabetes [31]. This pattern is consistent with known epidemiology: T2DM and insulin resistance are established risk factors for colorectal neoplasia [1], whereas diabetes appears inversely associated with prostate cancer incidence. Meta-analyses indicate diabetic men have about a 9% lower risk of prostate cancer than non-diabetics, possibly due to hormonal alterations (e.g., lower testosterone or hyperinsulinemia’s growth-suppressive effects on the prostate) [32]. Our finding of fewer diabetics among prostate cases aligns with this attenuated relationship. Similarly, the higher proportion of people with diabetes in colorectal and hepatobiliary cancers in our cohort reflects metabolic syndrome’s impact on those tumours (all four patients with diabetes and liver tumours had underlying metabolic risk factors). Although our sample size for each cancer type was limited, the overall distribution of diabetes across tumour sites in our study mirrors these broader epidemiological associations.

The co-occurrence of cancer and T2DM has important prognostic implications. Diabetes is an independent adverse prognostic factor in oncology outcomes [33]. A landmark meta-analysis of 23 studies by Barone et al. found that pre-existing diabetes was associated with a 41% higher risk of all-cause mortality in cancer patients [3]. More recent data continue to demonstrate this survival gap. For instance, a 2025 multinational cohort study (across 7 European countries) reported that having T2DM at cancer diagnosis confers a 25% increase in overall mortality risk (hazard ratio ~1.25) even after adjusting for other factors. Cancer-specific mortality is also modestly higher (HR ≈ 1.13) in patients with diabetes. The survival disadvantage was evident even in certain less common cancers (e.g., bladder, ovarian) in that cohort [30]. The reasons are multifactorial: diabetes-related organ damage (cardiac, renal) may reduce tolerance to cancer therapies; chronic hyperglycaemia can impair the immune response and wound healing; and diabetes increases susceptibility to infections and treatment complications [34]. Our study did not directly measure outcomes, but these findings underscore that cancer patients with T2DM represent a high-risk population. They often require more intensive medical management—indeed, prior research shows such patients have higher unplanned hospitalisation rates and more extended hospital stays during cancer treatment. One study noted that among cancer patients with comorbid conditions, diabetes was the leading cause of emergency department revisits (24% of unplanned returns), and diabetic cancer patients undergoing surgery (e.g., for colorectal cancer) had post-operative hospital stays ~3 days longer than non-diabetics [1]. These real-world data highlight that the intersection of cancer and diabetes adds complexity to care and can worsen short-term and long-term outcomes. Aggressive management of glycemia and comorbid conditions is therefore essential in this group. Encouragingly, experts advise that clinicians actively optimise cardiometabolic comorbidities in cancer patients to improve survival. Our findings reinforce this need, especially in tumour types like colorectal or liver cancer, where metabolic risk modification might impact cancer prognosis as well.

### 4.2. Ecog Performance Status in Patients with and Without T2DM

We examined whether diabetic patients were presented with worse functional status (ECOG performance score) than their non-diabetic counterparts. Contrary to our initial hypothesis, there was no significant difference—the mean ECOG score was virtually identical (~2.5 in both groups)—and the proportion of patients with an ECOG score of ≥3 was approximately 55% in both the diabetic and non-diabetic groups. In other words, having T2DM did not correlate with worse performance status at presentation in our cohort. This finding suggests that the severity of the cancer itself was the dominant determinant of functional status, overshadowing any incremental frailty from diabetes [35]. Many non-diabetic patients were profoundly debilitated by advanced malignancies (over half of our total sample had ECOG 3–4), which likely “levelled the playing field.” [36]. It appears that, by the time patients required radiotherapy or oncology admission, cancer-related factors (tumour burden, cachexia, pain) drove their performance status more than chronic conditions like diabetes. Another consideration is that our diabetic subgroup, though small (n = 20), was relatively well-managed—most were on oral hypoglycaemics or diet control, and none had acute hyperglycaemic crises or uncontrolled comorbidities during treatment. Effective outpatient diabetes management might have mitigated potential functional decline. Additionally, the people with diabetes in our study were of a similar age to those without diabetes, so age was not a confounding factor for the differences in performance status.

It is worth noting that comorbidity and performance status are often interrelated in cancer patients, even if we did not observe a direct diabetes effect. Patients with multiple chronic diseases tend to have lower baseline functional reserve and may tolerate cancer treatments poorly [30]. Prior studies have shown that comorbid conditions can lead to less aggressive cancer therapy being offered (for fear of toxicity), indirectly affecting outcomes [37]. In our cohort, however, treatment intensity (e.g., curative chemoradiation for cervical cancer, multimodal therapy for head/neck cancer) appeared to be driven by tumour factors and patient preference rather than diabetes status—people with diabetes were not generally undertreated. Our findings align with a recent analysis, which found that a history of diabetes did not significantly alter healthcare utilisation among cancer patients when adjusting for other factors [1]. Nonetheless, we remain cautious in interpreting the lack of ECOG difference: it may simply reflect that cancer’s impact on functional status is so severe in an oncology referral population that additional deficits from diabetes are marginal. Although our sample did not show statistically significant differences, it is plausible, based on prior reports, that larger or more diverse samples might reveal such distinctions.

We also assessed ECOG at a single time point (presentation to radiotherapy). Diabetes could still influence longitudinal functional decline or recovery trajectories. For instance, one might hypothesise that people with diabetes experience slower recovery from treatment or more fatigue over time due to metabolic issues, questions that warrant prospective follow-up. Overall, our data suggest that having T2DM should not be viewed as a barrier to aggressive cancer therapy based on performance status alone, since people with diabetes were just as likely as others to have good functional status if their cancer was not advanced. It reinforces the importance of individualised assessment; chronologic age or comorbidities alone should not prejudge a patient’s fitness for treatment, in line with geriatric oncology principles.

Importantly, the relationship between depression, T2DM, and cancer may be bidirectional. While our primary focus was on how chronic illness contributes to psychological distress, increasing evidence suggests the reverse is also true: that depression can predispose individuals to metabolic and oncologic disease via inflammatory mechanisms. Major depressive disorder is associated with HPA axis dysregulation, elevated cortisol, and altered autonomic function, all of which contribute to immune dysfunction and chronic low-grade inflammation. Increased circulating levels of IL-6, CRP, and TNF-α have been reported in depressed patients, even in the absence of overt medical illness. These mediators may impair insulin sensitivity and promote oncogenic processes, including angiogenesis and resistance to apoptosis. Thus, psychological distress may not only result from chronic disease but may also contribute to its development or progression. This underscores the importance of early psychiatric assessment—not only to enhance quality of life, but also as a potentially preventive intervention.

### 4.3. High Prevalence of Psychiatric Comorbidity and Influence of Diabetes

In our cohort, 58% of patients had clinically documented anxiety and/or depression, reflecting the high psychological toll of cancer. This prevalence lies at the upper end of ranges reported in the literature. Population studies generally indicate that 20–50% of cancer patients suffer from significant depression or anxiety, varying by cancer type and assessment method. For example, depression rates of 33–50% have been noted in pancreatic cancer and 22–57% in head and neck cancers—cancers known for high symptom burden—whereas some other tumours have lower reported rates [38]. Our cohort’s 58% likely reflects several factors: we included any clinically significant distress (not only major depressive disorder), but our centre also has a proactive liaison psychiatry programme (perhaps increasing recognition/documentation of psych symptoms). A large proportion of our patients had advanced disease (which is associated with more distress). Indeed, we observed that certain subgroups—notably head/neck cancer patients facing disfigurement and cervical cancer patients who were often younger women with family and social stressors—had exceptionally high rates of anxiety/depression, consistent with trends reported in psycho-oncology studies.

These results confirm the high prevalence of psychiatric symptoms—especially depression and anxiety—in cancer patients, regardless of diabetes status. The psychological burden appears more closely related to cancer localisation and ECOG functional status than to metabolic comorbidity.

Given the high burden of psychiatric symptoms observed, our findings underscore the value of integrating early psychosocial screening into oncology workflows. This is consistent with recommendations from integrative care models and supports the need for multidisciplinary approaches in oncology practice.

When comparing psychiatric comorbidity between those with and without T2DM, we noted a numerical trend toward more frequent anxiety/depression in the diabetic subgroup (70% vs. 56.5%), but this difference was not statistically significant. The odds of having a recorded psychiatric diagnosis were about 1.8-fold higher in diabetics, but with a wide confidence interval that crossed 1. In comparison, our diabetic patients did exhibit somewhat greater psychosocial distress, but the small diabetic subsample (n = 20) limited statistical power.

Epidemiological data from general medical populations show that diabetes is strongly associated with depression, with approximately 10–12% higher prevalence of depressive symptoms compared to the non-diabetic population [15]. This bidirectional relationship may arise from behavioural stressors related to diabetes management and the neurobiological effects of hyperglycaemia. However, in oncology, baseline distress is elevated due to the emotional impact of cancer itself, potentially masking additional effects of comorbidities like T2DM.

Our findings support this interpretation. Despite the lack of statistical significance, all diabetic patients with rectal cancer in our sample reported substantial depressive or anxious symptoms, often related to the dual burden of cancer and colostomy management atop diabetes care. In contrast, some non-diabetic rectal cancer patients coped more effectively. We also documented cases of panic attacks during radiotherapy sessions in diabetic patients, which were not observed in non-diabetics.

We anecdotally observed that diabetic patients, particularly those with rectal cancer, often experienced more intense psychological distress, including panic episodes. Although these patterns did not reach statistical significance, they align with qualitative literature suggesting that cancer patients with diabetes may face heightened psychological burden, especially when treatment complexity intersects with diabetes self-management. This trend may reflect both behavioural stress and physiological vulnerability, as discussed in psycho-oncology literature.

This is consistent with previous reports showing worse quality of life in cancer patients with comorbid diabetes, particularly if their glycaemic control is suboptimal during treatment [39]. For example, Kern et al. documented heightened anxiety and frustration in diabetic breast cancer survivors who had to manage blood sugars during chemotherapy [39,40]. Our findings underscore the importance of tailored psychosocial interventions in oncology, particularly for patients facing multiple health-related challenges. While T2DM did not statistically increase the incidence of psychiatric symptoms in our sample, these patients may still warrant extra attention.

### 4.4. Psychological Distress Severity (Hads Scores) and Correlation with Ecog

To quantify psychological symptoms, we analysed Hospital Anxiety and Depression Scale (HADS) questionnaires from a subset of 136 patients. The results painted a sobering picture: overall, the mean scores for both anxiety and depression subscales were around 15–16 (out of 21), significantly above the usual clinical cut-off of 11. Indeed, over 80% of patients who completed HADS met the criteria for at least moderate anxiety or depression. These figures confirm that most of our patients were experiencing clinically significant distress, supporting the high prevalence noted in chart diagnoses. When stratified by diabetes status, there were no significant differences in mean HADS scores—in fact, diabetic patients had slightly lower average anxiety scores (14.8 vs. 16.1), with similar depression scores compared to non-diabetics. This somewhat counterintuitive finding (one might expect people with diabetes to have higher distress) can be explained by a few considerations. First, as discussed, cancer-related distress was already widespread in both groups, potentially overshadowing any modest increase due to diabetes. Second, diabetic patients were on average slightly older (though not significantly), and we observed anecdotally that older patients tended to report slightly less anxiety, possibly due to better emotional coping or acceptance, or different response styles (younger patients sometimes had more overt anxiety about family and the future). Third, some diabetic patients might have already been receiving psychiatric treatment (e.g., SSRIs or anxiolytics) before or during cancer care, which could have reduced their reported anxiety levels. Regardless, the absence of a diabetes effect on HADS reinforces our earlier point: the presence of cancer created a uniformly high distress environment, so having T2DM did not significantly worsen self-reported mood symptoms. This aligns with the non-significant difference in diagnosed psychiatric comorbidity rates. It also echoes findings from a supplementary analysis we conducted using HADS cut-offs: the proportions of patients with HADS-Anxiety scores ≥ 11 was 75% (diabetic) versus 82% (non-diabetic), *p* = 0.49, and for HADS-Depression scores ≥ 11, it was 75% versus 86%, *p* = 0.28. Thus, distress was widespread in both groups, and diabetes was not a key factor in our quantitative mental health assessment.

One of the most salient findings in our study was the relationship between performance status and psychological distress. We found a moderate, statistically significant positive correlation between ECOG score and HADS total score (Spearman ρ = 0.414, *p* < 0.001), indicating that patients with worse functional status (higher ECOG) tended to report higher levels of combined anxiety/depression [41]. In practical terms, those who were more debilitated by their cancer also felt more psychologically distressed. This correlation is intuitive and supported by wider literature [20,42]. Advanced disease often brings greater symptom burden (pain, fatigue, disability), which can precipitate depression and anxiety. Conversely, patients who are very depressed may be less active or perceive their functional status as lower. Our objective ECOG assessments align with the notion that physical and psychological well-being decline in tandem. In our data, the mean HADS scores were lowest in the few patients with an ECOG score of 1 and progressively higher in those with ECOG scores of 2, 3, and 4. For instance, mean HADS-Depression was ~13.6 at ECOG 1 and ~17–19 at ECOG 3–4, indicating a marked increase in depressive symptoms in those with significant functional impairment. This trend remained evident even when we dichotomised: patients with “good” performance (ECOG 0–2) were much more likely to have normal HADS scores, whereas those with ECOG 3–4 almost universally had abnormal (elevated) HADS scores. A recent study of advanced lung cancer patients similarly reported that those with ECOG 3–4 and inpatients/palliative care settings had very high anxiety and depression levels, whereas patients with ECOG 0–1 or on outpatient treatment had significantly lower distress. The authors noted that hopelessness and loss of autonomy in those with poor performance status contributed to psychological suffering [43]. Our findings are consonant with this: for many patients, declining functional status likely means loss of independence, increased pain/fatigue, and a sense of approaching end-of-life—all of which fuel depression and anxiety. From a biological perspective, this link may also be mediated by disease severity: patients with worse ECOG often have more aggressive or widespread cancer, which is frequently accompanied by higher levels of inflammatory cytokines (e.g., IL-6, TNF-α) and paraneoplastic symptoms that can induce “sickness behaviour” (lethargy, low mood) [44]. Clinical vigilance is therefore required—patients with poor ECOG should be routinely screened and treated for depression/anxiety, as addressing their mental health needs is integral to palliation. Conversely, helping to manage distress (through counselling, medications, physical therapy for symptom control, etc.) might in turn improve patients’ functional ability or at least their perception of it. Our study underscores the importance of this mind–body interplay: treating the whole patient is crucial, as psychological and physical states are deeply interconnected [43,45,46].

### 4.5. Multivariable Analyses and Robustness Checks

Our adjusted analyses with N = 136 confirmed that ECOG performance status remained independently associated with both depressive and anxiety symptoms, consistent with univariate findings. Conversely, T2DM showed no independent association with HADS scores or with the odds of ECOG ≥ 3 after adjustment for age, sex, and residence. Interestingly, older age was inversely associated with anxiety levels, suggesting possible resilience or cohort effects. These findings underscore that functional decline is a stronger correlate of distress than diabetes comorbidity. Similar observations have been reported in oncology cohorts, where ECOG was an independent predictor of anxiety and depression after controlling for illness severity [47,48,49,50].

### 4.6. Neuroinflammatory Links Between Cancer, Diabetes, and Depression

Although our study did not include direct measurements of inflammatory biomarkers, the observed clinical patterns are discussed here within a theoretical framework of neuroinflammation. This model is supported by converging evidence from prior literature, suggesting that chronic low-grade inflammation plays a central role in the pathogenesis of cancer, diabetes, and depression. These data reinforce

A growing body of evidence indicates that chronic low-grade inflammation is a common denominator in T2DM and major depressive disorder. Patients with either condition alone show elevated circulating proinflammatory cytokines such as interleukin-6 (IL-6) and tumour necrosis factor-alpha (TNF-α), among others. In diabetes, adipose tissue inflammation and high serum IL-6/TNF levels are well-documented, contributing to insulin resistance and also potentially affecting the brain and behaviour [15]. Cancer, especially advanced cancer, further amplifies this inflammatory milieu: tumours secrete cytokines (such as IL-1β, IL-6, and TNF-α) as part of immune evasion and tumour progression processes. These cytokines can cross the blood–brain barrier or activate vagal and microglial pathways, inducing central inflammatory responses that manifest as depressive symptoms (the so-called “sickness behaviour” syndrome). Notably, IL-6 has been implicated as a key mediator of cancer-related depression. For example, Lutgendorf et al. demonstrated in ovarian cancer patients that elevated IL-6 (in both plasma and even tumour ascites) correlated specifically with vegetative depressive symptoms like fatigue and anorexia [51,52]. Similarly, a 2022 meta-analysis by McFarland et al. found that among cancer patients, those with higher peripheral IL-6 and TNF-α levels had significantly worse depression scores (pooled effect sizes ~0.6–0.7). These data reinforce that inflammation is not just an epiphenomenon but a driving factor in depressive symptoms in oncology [53].

It is essential to emphasise that these insights are based on previous research, rather than being directly evaluated in our cohort. Therefore, the neuroinflammatory model we propose remains a conceptual framework for understanding the high levels of distress observed in this medically complex population.

In our study, while we did not measure cytokines, the clinical outcomes align with an inflammatory model. The fact that virtually all our patients—diabetic or not—had elevated distress suggests a common biological stressor, likely the cancer itself, with its inflammatory burden. Adding diabetes on top might raise inflammation further, but in an already high-inflammation environment (advanced cancer), the incremental effect may be small or hard to discern clinically [54]. It is plausible that both our diabetic and non-diabetic cancer patients had high IL-6/TNF due to cancer; the people with diabetes might have had slightly higher levels, but all above a threshold that predisposes to depression [55,56]. This could explain why we did not see significant mood differences by diabetes status: both groups had cytokine-driven depression to a similar degree. That said, one might speculate that specific inflammatory markers (like C-reactive protein or IL-6) were even more elevated in the diabetic subgroup, potentially contributing to the trend toward higher psychiatric morbidity. This is an area for future investigation—profiling inflammatory biomarkers in cancer patients with vs. without T2DM could elucidate whether the “double inflammation” of cancer + diabetes translates to greater neuroimmune activation. If so, it raises the question of therapeutic targeting. There is growing interest in using anti-inflammatory strategies to treat depression, especially in those with elevated inflammatory markers. Small trials of cytokine inhibitors (e.g., anti-TNF agents or IL-6 blockers) in treatment-resistant depression have shown modest benefits. In cancer patients, the use of anti-cytokine therapies (like tocilizumab for cancer-related cachexia or depression) is still experimental, but conceptually promising. Likewise, integrative interventions such as exercise and diet, which reduce systemic inflammation, could have dual benefits for glycaemic control and mood. Recent data also indicate that unhealthy behaviours—notably higher alcohol intake—are linked to heightened anxiety, depression and impulsivity even in otherwise healthy young adults, underscoring alcohol reduction as an additional modifiable target in neuroinflammation-driven mood disorders [57]. Our study’s context—a Special Issue on neuroinflammation in psychiatric conditions—underscores that tackling inflammation might be a unifying approach to improve outcomes in patients burdened with both medical and psychiatric illness. In summary, the interplay of cancer, diabetes, and depression can be viewed as a vicious cycle mediated in part by inflammatory cytokines and stress hormones. Breaking this cycle will likely require interventions that address both the biological underpinnings (inflammation, HPA axis dysregulation) and the psychosocial stressors [15,58].

In cancer patients, this inflammatory environment is often worsened by tumour-derived cytokines and treatment-related stressors. Notably, IL-6 has become a potential biomarker for cancer-related depression, especially in patients with advanced or treatment-resistant disease. Recent studies showed that baseline CRP and IL-6 levels predicted depressive symptoms during chemotherapy in a prospective cohort of lung cancer patients. These findings support the idea that neuroinflammation may be a shared underlying cause, possibly explaining the high rates of anxiety and depression seen in oncologic patients with metabolic comorbidities [51,59,60,61]. Our data supports this idea, although we did not directly test it.

### 4.7. Clinical and Public Health Implications: Toward Multidisciplinary Care

Our findings carry several clinical and public health implications. They highlight the need for a genuinely multidisciplinary approach in managing patients who stand at the crossroads of oncology, endocrinology, and psychiatry. First, from a clinical practice standpoint, the data support current guidelines that recommend integrated psychosocial and medical care in oncology. The American Society of Clinical Oncology (ASCO) and other bodies recommend routine distress screening for cancer patients and timely intervention (e.g., psycho-oncology referrals, counselling) as part of standard care. Our observation that over half of patients have significant anxiety/depression, and virtually all patients with poor performance status do, means that oncologists and nurses must be vigilant in assessing mental health at each stage. Embedding mental health professionals in oncology clinics or establishing referral pathways can ensure issues are addressed early. There is evidence that doing so improves outcomes: psychological interventions (such as cognitive-behavioural therapy, relaxation training, etc.) significantly enhance quality of life for cancer patients and can reduce distress levels by moderate effect sizes. While these interventions may not extend survival in early-stage cancers, they undeniably improve patients’ day-to-day well-being and emotional functioning, outcomes that are valuable in their own right and may aid patients in adhering to treatment plans [62,63].

Second, our study underscores the importance of co-managing metabolic comorbidities like diabetes during cancer treatment. In busy oncology practices, it is easy to focus narrowly on cancer and inadvertently neglect other chronic conditions. However, our findings and the literature suggest this would be a mistake: uncontrolled diabetes can lead to treatment interruptions (e.g., from infections or severe fatigue) and may worsen cancer prognosis [1]. Multidisciplinary case conferences, also known as “tumour boards,” should ideally include consideration of comorbid conditions—for example, involving an endocrinologist or internist when formulating a treatment plan for a patient with active diabetes. Some cancer centres have begun to develop “oncodiabetology” services, recognising the need for endocrinology input in patients receiving corticosteroids or other treatments that disrupt glucose control [64]. A recent national study highlighted that cancer patients often receive fragmented care: during active treatment, visits to primary care providers drop, and oncologists may not manage diabetes actively [39]. Interventions are needed to bridge this gap, whether through the co-location of services, telemedicine follow-ups for diabetes management, or clear protocols for comorbidity management. From a policy perspective, improving care coordination is paramount. Jo et al. found that patients with cancer and prediabetes had significantly higher healthcare utilisation (more visits) than those with cancer alone, suggesting that identifying and managing early deglycation could reduce downstream complications. They concluded that optimal care coordination and early metabolic risk management may improve cancer survivorship and reduce healthcare burden [1]. This supports initiatives to integrate primary care and oncology for survivorship, as well as during acute cancer care.

Third, our results have implications for resource allocation and survivor follow-up. As more patients survive longer with cancer, the convergence of cancer, diabetes, and depression will become increasingly prevalent. This triple comorbidity can be viewed as a “syndemic”—diseases that act synergistically to worsen overall patient health. Public health programmes should consider screening cancer survivors for metabolic syndrome and mental health problems as part of routine survivorship care. For example, a survivorship clinic visit might include HbA1c testing and a depression screen (HADS or PHQ-9), with referrals made accordingly. On the flip side, diabetologists should be aware that their patients have an elevated risk of certain cancers and depression and ensure appropriate cancer screenings and mental health evaluations are performed [12,65]. The need for integrative care models is evident. Multidisciplinary clinics or at least multi-speciality collaboration can yield better outcomes. In our local context, we found value in having a liaison psychiatrist and a nutrition/metabolic specialist as part of the oncology team. These inputs helped tailor interventions (like prescribing appetite stimulants or anxiolytics, adjusting diabetic diets during treatment) that improved patients’ tolerance of therapy. Patients themselves appreciate this approach: qualitative research reports that cancer patients with comorbid diabetes desire a designated provider or team to help coordinate their dual care, along with better education on how to manage diabetes during chemotherapy [65]. In Kern et al.’s study, participants suggested having a single care plan that addresses both illnesses, rather than siloed advice from oncologists and primary care [39,66]. This kind of patient-centred integration could reduce confusion and anxiety, thereby improving self-management. Our findings echo that sentiment—the more we treat the patient holistically, the better the likely outcomes in all domains.

The synergistic relationship between physical comorbidities and psychological distress in oncology demands an integrative management strategy. Cancer care must evolve from a single-disease model to a comprehensive model that accounts for metabolic health and mental health. Practically, this could involve routine distress thermometer screenings, endocrinology consults for hyperglycaemia management, physical rehabilitation to maintain function, and anti-inflammatory lifestyle interventions—all alongside standard oncologic treatment. Such multidisciplinary care not only addresses current needs but may also confer long-term benefits, as improved metabolic and mental health could potentially translate into better cancer survival [30]. Notably, our study provides evidence from an Eastern European population, a group underrepresented in the psycho-oncology literature, reinforcing that these issues transcend geographic and healthcare system differences. The challenge ahead lies in implementation—aligning specialists, training oncology providers in basic diabetes/psychiatric care (and vice versa for primary care providers in cancer survivorship), and ensuring patients do not “fall through the cracks” between specialities. The public health stake is high: with millions of cancer survivors worldwide, many of whom carry comorbid conditions, investing in integrative care models is investing in improved quality of life and outcomes for a growing segment of our population.

Our findings support the idea that psychological distress is widespread among cancer patients, regardless of diabetes status, but the additional challenge of managing coexisting T2DM may require more careful attention. Interventions that target IL-6, oxidative stress, or microglial activation could serve as promising translational strategies to reduce both psychiatric symptoms and systemic disease progression. Future research should explore biomarkers of neuroinflammation (e.g., CRP, IL-6, kynurenine pathway metabolites) to better define risk profiles and tailor supportive care.

This study has several limitations. First, the diabetic subgroup was small (n = 20), which limits statistical power and precludes strong conclusions about diabetes-specific effects. Second, HADS data were available only for 136 of the 174 patients, which may introduce selection bias. Patients without psychological assessments may differ systematically from those included in the analysis, for example, in terms of clinical severity or timing of evaluation. Third, the single-centre design and inclusion of patients with advanced-stage cancers limit the generalizability of the results. Furthermore, neuroinflammation was discussed as a theoretical framework but was not directly assessed in this study.

Additionally, due to the specific profile of the radiotherapy centre, there was an overrepresentation of pelvic and head and neck cancers, while certain tumour types (e.g., hematologic or early-stage breast cancers) were underrepresented, further limiting the generalizability of findings to the broader cancer population.

Beyond the cross-sectional design, multivariable analyses were limited by the small number of T2DM cases and incomplete tumour-site data, which widened confidence intervals and increased the risk of a type-II error. Although robust errors and sensitivity assessments were conducted, residual confounding cannot be ruled out. Logistic modelling of psychiatric comorbidity (HADS ≥ 11) was constrained by quasi-separation due to high prevalence.

Although our findings showed a slightly higher percentage of psychiatric diagnoses in patients with T2DM, HADS scores did not differ significantly between groups. This indicates that the distress burden was primarily driven by the cancer diagnosis itself, rather than by metabolic status. As such, we emphasise that these differences are descriptive rather than inferential.

## 5. Conclusions

In this retrospective cohort of Romanian cancer patients, we found a high prevalence of psychiatric comorbidity, with nearly 60% of patients presenting clinically significant anxiety or depression. Psychological distress was similarly frequent among patients with and without type 2 diabetes mellitus (T2DM), indicating that cancer itself remains the predominant driver of mental health burden.

Functional impairment, measured by ECOG performance status, was strongly correlated with both anxiety and depression scores. Patients with poorer performance status (ECOG ≥ 3) consistently reported higher levels of distress, underscoring the close link between physical debility and psychological suffering in oncology.

These findings highlight the importance of routine psychiatric screening and early psychosocial intervention for all cancer patients, regardless of metabolic status. Multidisciplinary care models that integrate oncology, endocrinology, and psycho-oncology are recommended to improve both quality of life and treatment adherence.

Future research should address several directions. First, longitudinal and multicentre designs are needed to capture trajectories of functional decline and psychological distress in cancer patients with and without T2DM, moving beyond cross-sectional associations. Second, integration of objective biomarkers of inflammation (e.g., CRP, IL-6, TNF-α) could help clarify mechanistic pathways linking metabolic disease and psychiatric comorbidity. Third, comparative effectiveness studies may identify whether targeted interventions—such as early psychiatric screening, structured diabetes management, or anti-inflammatory approaches—improve treatment tolerance and outcomes. Finally, extending research to broader Eastern European populations would provide more representative regional evidence and enhance generalizability.

## Figures and Tables

**Table 1:** Distribution of cancer types.

Cancer Type	N	%
**Cervical**	41	23.6%
**Head and Neck**	34	19.5%
**Breast**	26	14.9%
**Rectal**	20	11.5%
**Lung**	15	8.6%
**Oesophageal**	9	5.2%
**Prostate**	8	4.6%
**Bladder**	6	3.4%
**Other/Misc.**	15	8.6%
**Total**	174	100%

**Table 2:** Baseline characteristics.

Characteristic	All Patients (N = 174)	Non-T2DM (n = 154)	T2DM (n = 20)
Age, years (mean ± SD)	68.1 ± 9.9	67.9 ± 10.1	69.5 ± 8.4
Sex, Male (%)	85 (48.9%)	77 (50.0%)	8 (40.0%)
Urban Residence (%)	84 (76.4%) *	seventy (–)	twelve (–)
ECOG Performance (mean ± SD)	2.53 ± 0.74	2.54 ± 0.75	2.50 ± 0.76
ECOG ≥ 3 (%)	96 (55.2%)	85 (55.2%)	11 (55.0%)
Psychiatric Comorbidity (%)	101 (58.0%)	87 (56.5%)	14 (70.0%)
HADS Anxiety (mean ± SD)	15.9 ± 4.1 ‡	16.1 ± 4.2	14.8 ± 3.2
HADS Depression (mean ± SD)	16.3 ± 4.0 ‡	16.4 ± 4.1	15.6 ± 3.5

Abbreviations: SD—standard deviation; ECOG—Eastern Cooperative Oncology Group. Psychiatric comorbidity includes any documented anxiety and/or depressive syndrome. HADS, Hospital Anxiety Depression Scale (scores range from 0 to 21 for each subscale); T2DM, type 2 diabetes mellitus; non-T2DM, patients without T2DM. * Residence data available for 110 patients (84 urban, 26 rural); remaining not recorded. ‡ HADS data available for 136 patients (110 non-T2DM, 16 T2DM). This study adhered to the Strengthening the Reporting of Observational Studies in Epidemiology (STROBE) guidelines for observational research.

**Table 3:** Descriptive statistics of HADS scores by ECOG performance status.

Measure (HADS Subscale)	ECOG	N	Mean	Median	SD	Minimum	Maximum
HADS Anxiety	1	8	15.8	16.0	4.06	10.00	21.0
	2	44	15.0	14.5	3.33	8.00	21.0
	3	77	16.2	17.0	3.07	9.00	21.0
	4	6	19.0	19.0	1.41	17.00	21.0
HADS Depression	1	8	13.6	13.5	1.77	12.00	17.0
	2	44	14.7	14.0	2.96	10.00	21.0
	3	77	17.2	18.0	2.65	10.00	21.0
	4	6	19.5	19.5	1.05	18.00	21.0
HADS Total	1	8	29.4	29.5	5.29	22.00	38.0
	2	44	29.7	30.0	4.75	20.00	41.0
	3	77	33.4	35.0	5.00	23.00	41.0
	4	6	38.5	38.0	2.17	36.00	41.0

**Table 4:** Spearman’s correlation between ECOG performance status and total psychological distress (HADS Total). n = 135; ρ = 0.414, *p* < 0.001.

Test	Variable	Statistic	df	*p*-Value	Effect Size
ANOVA	HADS Total	F = 9.81	3, 131	<0.001	η^2^ = 0.184
Kruskal–Wallis	HADS Anxiety	χ^2^ = 10.0	3	0.018	ε^2^ = 0.075
	HADS Depression	χ^2^ = 31.3	3	<0.001	ε^2^ = 0.234
	HADS Total	χ^2^ = 25.0	3	<0.001	ε^2^ = 0.186

**Table 5:** Games–Howell pairwise comparisons for HADS-Total by ECOG performance status (N = 135).

Comparison	Mean Difference (HADS Total)	*p*-Value
ECOG 1 vs. 2	−0.28	0.999
ECOG 1 vs. 3	−4.01	0.243
ECOG 1 vs. 4	−9.13	0.006 *
ECOG 2 vs. 3	−3.73	<0.001 *
ECOG 2 vs. 4	−8.84	<0.001 *
ECOG 3 vs. 4	−5.11	0.003 *

* *p* < 0.05 considered statistically significant.

**Table 6:** Multivariable linear regression for HADS-Depression (N = 136; OLS with HC3 SEs).

Predictor	β	SE	95% CI	*p*
ECOG (per 1-point increase)	**2.212**	0.319	[1.586, 2.838]	**<0.001**
T2DM (yes vs. no)	−0.555	0.790	[−2.103, 0.993]	0.482
Age (per 1-year increase)	0.018	0.034	[−0.048, 0.084]	0.589
Female (vs. male)	**0.774**	0.474	[−0.154, 1.701]	**0.102**
Rural (vs. urban)	−1.026	0.813	[−2.618, 0.565]	0.207

**Table 7:** Multivariable logistic regression for ECOG ≥ 3 (N = 136).

Predictor	aOR	95% CI	*p*
T2DM (yes vs. no)	**0.587**	0.204–1.688	0.323
Age (per 1 y ↑)	0.998	0.948–1.051	0.942
Female (vs. male)	0.601	0.283–1.278	0.186
Rural (vs. urban)	1.585	0.478–5.256	0.451

Notes: Logistic model adjusted for age, sex, and residence; aOR = adjusted odds ratio.

## Data Availability

The datasets generated and analysed during the current study are available from the corresponding author on reasonable request. Data are not publicly available due to privacy restrictions.

## Abbreviations

The following abbreviations are used in this manuscript:

T2DM	Type 2 diabetes mellitus
ECOG	Eastern Cooperative Oncology Group
HADS	Hospital Anxiety Depression Scale
ASCO	American Society of Clinical Oncology
ICD-10	International Classification of Diseases, 10th Revision
STROBE	Strengthening the Reporting of Observational Studies in Epidemiology

## References

[1] Jo A. (2024). Health Care Use Among Cancer Patients With Diabetes, National Health and Nutrition Examination Survey, 2017–2020. Prev. Chronic Dis..

[2] Liang X.P., Or C.Y., Tsoi M.F., Cheung C.L., Cheung B.M.Y. (2021). Division of Clinical Pharmacology and Therapeutics, D. of M., The University of Hong Kong, Hong Kong Prevalence of Metabolic Syndrome in the United States National Health and Nutrition Examination Survey (Nhanes) 2011–2018. Eur. Heart J..

[3] Barone B.B., Yeh H.-C., Snyder C.F., Peairs K.S., Stein K.B., Derr R.L., Wolff A.C., Brancati F.L. (2008). Long-Term All-Cause Mortality in Cancer Patients With Preexisting Diabetes Mellitus: A Systematic Review and Meta-Analysis. JAMA.

[4] Kirschner M.B., Pulford E., Hoda M.A., Rozsas A., Griggs K., Cheng Y.Y., Edelman J.J.B., Kao S.C., Hyland R., Dong Y. (2015). Fibulin-3 Levels in Malignant Pleural Mesothelioma Are Associated with Prognosis but Not Diagnosis. Br. J. Cancer.

[5] Larsson S.C., Orsini N., Wolk A. (2005). Diabetes Mellitus and Risk of Colorectal Cancer: A Meta-Analysis. J. Natl. Cancer Inst..

[6] Tsilidis K.K., Kasimis J.C., Lopez D.S., Ntzani E.E., Ioannidis J.P.A. (2015). Type 2 Diabetes and Cancer: Umbrella Review of Meta-Analyses of Observational Studies. BMJ.

[7] Giovannucci E., Harlan D.M., Archer M.C., Bergenstal R.M., Gapstur S.M., Habel L.A., Pollak M., Regensteiner J.G., Yee D. (2010). Diabetes and Cancer: A Consensus Report. Diabetes Care.

[8] Kapoor A., Hornig M., Asokan A., Williams B., Henriquez J.A., Lipkin W.I. (2011). Bocavirus Episome in Infected Human Tissue Contains Non-Identical Termini. PLoS ONE.

[9] Pâslaru A.-C., Călin A., Morozan V.-P., Stancu M., Tofan L., Panaitescu A.M., Zăgrean A.-M., Zăgrean L., Moldovan M. (2024). Burst-Suppression EEG Reactivity to Photic Stimulation—A Translational Biomarker in Hypoxic–Ischemic Brain Injury. Biomolecules.

[10] Niedzwiedz C.L., Knifton L., Robb K.A., Katikireddi S.V., Smith D.J. (2019). Depression and Anxiety among People Living with and beyond Cancer: A Growing Clinical and Research Priority. BMC Cancer.

[11] Grassi L., Caruso R., Riba M.B., Lloyd-Williams M., Kissane D., Rodin G., McFarland D., Campos-Ródenas R., Zachariae R., Santini D. (2023). Anxiety and Depression in Adult Cancer Patients: ESMO Clinical Practice Guideline†. ESMO Open.

[12] Koyama A.K. (2023). State-Specific Prevalence of Depression Among Adults With and Without Diabetes—United States, 2011–2019. Prev. Chronic Dis..

[13] Wicke F.S., Otten D., Schulz A., Wild P.S., Lackner K.J., Münzel T., König J., Ernst M., Wiltink J., Reiner I. (2024). Current and Past Depression as Risk Factors for Incident Type 2 Diabetes Mellitus and Pre-Diabetes in Men and Women: Evidence from a Longitudinal Community Cohort. Diabetol. Metab. Syndr..

[14] Macciò A., Madeddu C., Macciò A., Madeddu C. (2012). Inflammation and Ovarian Cancer. Ovarian Cancer—Basic Science Perspective.

[15] Bodnaruc A.M., Roberge M., Giroux I., Aguer C. (2024). The Bidirectional Link between Major Depressive Disorder and Type 2 Diabetes: The Role of Inflammation. Endocrines.

[16] Avram O.-E., Bratu E.-A., Curis C., Moroianu L.-A., Drima E. (2025). Modifiable Nutritional Biomarkers in Autism Spectrum Disorder: A Systematic Review and Meta-Analysis of Vitamin D, B12, and Homocysteine Exposure Spanning Prenatal Development Through Late Adolescence. Int. J. Mol. Sci..

[17] Dantzer R., O’Connor J.C., Freund G.G., Johnson R.W., Kelley K.W. (2008). From Inflammation to Sickness and Depression: When the Immune System Subjugates the Brain. Nat. Rev. Neurosci..

[18] McFarland D.C., Riba M., Grassi L. (2021). Clinical Implications of Cancer Related Inflammation and Depression: A Critical Review. Clin. Pract. Epidemiol. Ment. Health CP EMH.

[19] Dănăilă E., Bounegru I., Benea L., Chiriac A. (2014). Improving Biocompatibility of Co-Cr Alloy Used in Dentistry by Surface Modification with Electrochemical Methods—Corrosion of Untreated Co-Cr Alloy in Solution with Different pH. Ann. Dunarea De Jos Univ. Galati Fascicle IX Metall. Mater. Sci..

[20] Mitchell A.J., Chan M., Bhatti H., Halton M., Grassi L., Johansen C., Meader N. (2011). Prevalence of Depression, Anxiety, and Adjustment Disorder in Oncological, Haematological, and Palliative-Care Settings: A Meta-Analysis of 94 Interview-Based Studies. Lancet Oncol..

[21] Linden W., Vodermaier A., MacKenzie R., Greig D. (2012). Anxiety and Depression after Cancer Diagnosis: Prevalence Rates by Cancer Type, Gender, and Age. J. Affect. Disord..

[22] Anderson R.J., Freedland K.E., Clouse R.E., Lustman P.J. (2001). The Prevalence of Comorbid Depression in Adults With Diabetes: A Meta-Analysis. Diabetes Care.

[23] Carreira H., Williams R., Müller M., Harewood R., Stanway S., Bhaskaran K. (2018). Associations Between Breast Cancer Survivorship and Adverse Mental Health Outcomes: A Systematic Review. J. Natl. Cancer Inst..

[24] Lu Y., Tao J. (2021). Diabetes Mellitus and Obesity as Risk Factors for Bladder Cancer Prognosis: A Systematic Review and Meta-Analysis. Front. Endocrinol..

[25] Gonzalez J.S., Peyrot M., McCarl L.A., Collins E.M., Serpa L., Mimiaga M.J., Safren S.A. (2008). Depression and Diabetes Treatment Nonadherence: A Meta-Analysis. Diabetes Care.

[26] Stierman B., Afful J., Carroll M.D., Chen T.-C., Davy O., Fink S., Fryar C.D., Gu Q., Hales C.M., Hughes J.P. (2021). National Health and Nutrition Examination Survey 2017–March 2020 Prepandemic Data Files-Development of Files and Prevalence Estimates for Selected Health Outcomes. Natl. Health Stat. Report..

[27] Luo J., Lin H.-C., He K., Hendryx M. (2014). Diabetes and Prognosis in Older Persons with Colorectal Cancer. Br. J. Cancer.

[28] Ma S.J., Oladeru O.T., Singh A.K. (2020). Association of Survival With Chemoendocrine Therapy in Women With Small, Hormone Receptor–Positive, ERBB2-Positive, Node-Negative Breast Cancer. JAMA Netw. Open.

[29] Niculet E., Craescu M., Rebegea L., Bobeica C., Nastase F., Lupasteanu G., Stan D.J., Chioncel V., Anghel L., Lungu M. (2022). Basal Cell Carcinoma: Comprehensive Clinical and Histopathological Aspects, Novel Imaging Tools and Therapeutic Approaches (Review). Exp. Ther. Med..

[30] Davila-Batista V., Viallon V., Fontvieille E., Jansana A., Kohls M., Bondonno N.P., Tjønneland A., Dahm C.C., Antoniussen C.S., Katzke V. (2025). Associations between Cardiometabolic Comorbidities and Mortality in Adults with Cancer: Multinational Cohort Study. BMJ Med..

[31] Diabetes in America: Prevalence, Statistics, and Economic Impact. https://diabetes.org/about-diabetes/statistics/about-diabetes.

[32] Bonovas S., Filioussi K., Tsantes A. (2004). Diabetes Mellitus and Risk of Prostate Cancer: A Meta-Analysis. Diabetologia.

[33] Diabetes. https://www.who.int/news-room/fact-sheets/detail/diabetes.

[34] Zimmet P., Alberti K.G.M.M., Shaw J. (2001). Global and Societal Implications of the Diabetes Epidemic. Nature.

[35] Schnipper L.E., Smith T.J., Raghavan D., Blayney D.W., Ganz P.A., Mulvey T.M., Wollins D.S. (2012). American Society of Clinical Oncology Identifies Five Key Opportunities to Improve Care and Reduce Costs: The Top Five List for Oncology. J. Clin. Oncol..

[36] Extermann M., Hurria A. (2007). Comprehensive Geriatric Assessment for Older Patients With Cancer. J. Clin. Oncol..

[37] Shahid R.K., Ahmed S., Le D., Yadav S. (2021). Diabetes and Cancer: Risk, Challenges, Management and Outcomes. Cancers.

[38] Massie M.J. (2004). Prevalence of Depression in Patients With Cancer. JNCI Monogr..

[39] Pinheiro L.C., Cho J., Rothman J., Zeng C., Wilson M., Kern L.M., Tamimi R.M., Safford M.M. (2023). Diabetes and Cancer Co-Management: Patient-Reported Challenges, Needs, and Priorities. Support. Care Cancer.

[40] Kern E., Chun J., Schwartz S., Billig J., Friedman E., Eddy M., Kiely D., Guth A., Axelrod D., Schnabel F. (2014). The Breast Cancer Lifestyle Intervention Pilot Study. J. Cancer Ther..

[41] Temel J.S., Greer J.A., Muzikansky A., Gallagher E.R., Admane S., Jackson V.A., Dahlin C.M., Blinderman C.D., Jacobsen J., Pirl W.F. (2010). Early Palliative Care for Patients with Metastatic Non–Small-Cell Lung Cancer. N. Engl. J. Med..

[42] Mystakidou K., Tsilika E., Parpa E., Katsouda E., Galanos A., Vlahos L. (2005). Assessment of Anxiety and Depression in Advanced Cancer Patients and Their Relationship with Quality of Life. Qual. Life Res..

[43] Rahnea-Nita R.-A., Rebegea L.-F., Dumitru M., Mitrica R.-I., Nechifor A., Firescu D., Maier A.-C., Constantin G.B., Grigorean V.-T., Rahnea-Nita G. (2024). Anxiety and Depression in Advanced and Metastatic Lung Cancer Patients—Correlations with Performance Status and Type of Treatment. Medicina.

[44] Dantzer R., Meagher M.W., Cleeland C.S. (2012). Translational Approaches to Treatment-Induced Symptoms in Cancer Patients. Nat. Rev. Clin. Oncol..

[45] Holland J.C., Andersen B., Breitbart W.S., Buchmann L.O., Compas B., Deshields T.L., Dudley M.M., Fleishman S., Fulcher C.D., Greenberg D.B. (2013). Distress Management. J. Natl. Compr. Cancer Netw..

[46] Li M., Kennedy E.B., Byrne N., Gérin-Lajoie C., Katz M.R., Keshavarz H., Sellick S., Green E. (2016). Management of Depression in Patients With Cancer: A Clinical Practice Guideline. J. Oncol. Pract..

[47] Endeshaw D., Walle T.A., Yohannes S. (2022). Depression, Anxiety and Their Associated Factors among Patients with Cancer Receiving Treatment at Oncology Units in Amhara Region, Ethiopia: A Cross-Sectional Study. BMJ Open.

[48] Bužgová R., Jarošová D., Hajnová E. (2015). Assessing Anxiety and Depression with Respect to the Quality of Life in Cancer Inpatients Receiving Palliative Care. Eur. J. Oncol. Nurs..

[49] Hinz A., Herzberg P.Y., Lordick F., Weis J., Faller H., Brähler E., Härter M., Wegscheider K., Geue K., Mehnert A. (2019). Age and Gender Differences in Anxiety and Depression in Cancer Patients Compared with the General Population. Eur. J. Cancer Care.

[50] Vissers P.A.J., Falzon L., van de Poll-Franse L.V., Pouwer F., Thong M.S.Y. (2016). The Impact of Having Both Cancer and Diabetes on Patient-Reported Outcomes: A Systematic Review and Directions for Future Research. J. Cancer Surviv..

[51] Lutgendorf S.K., Weinrib A.Z., Penedo F., Russell D., DeGeest K., Costanzo E.S., Henderson P.J., Sephton S.E., Rohleder N., Lucci J.A. (2008). Interleukin-6, Cortisol, and Depressive Symptoms in Ovarian Cancer Patients. J. Clin. Oncol..

[52] Hanahan D., Weinberg R.A. (2011). Hallmarks of Cancer: The Next Generation. Cell.

[53] McFarland D.C., Doherty M., Atkinson T.M., O’Hanlon R., Breitbart W., Nelson C.J., Miller A.H. (2022). Cancer-Related Inflammation and Depressive Symptoms: Systematic Review and Meta-Analysis. Cancer.

[54] Roohi E., Jaafari N., Hashemian F. (2021). On Inflammatory Hypothesis of Depression: What Is the Role of IL-6 in the Middle of the Chaos?. J. Neuroinflammation.

[55] Yin Y., Ju T., Zeng D., Duan F., Zhu Y., Liu J., Li Y., Lu W. (2024). “Inflamed” Depression: A Review of the Interactions between Depression and Inflammation and Current Anti-Inflammatory Strategies for Depression. Pharmacol. Res..

[56] Osimo E.F., Pillinger T., Rodriguez I.M., Khandaker G.M., Pariante C.M., Howes O.D. (2020). Inflammatory Markers in Depression: A Meta-Analysis of Mean Differences and Variability in 5,166 Patients and 5,083 Controls. Brain Behav. Immun..

[57] Mitincu-Caramfil S.D., Moroianu L.-A., Bradeanu A.V., Isailă O.-M., Curis C., Drima E. (2025). The Correlation Between Emotionality Changes and Alcohol Consumption in Young Persons: A Pilot Study. Healthcare.

[58] Bounegru A.V., Bounegru I. (2024). Acrylamide in Food Products and the Role of Electrochemical Biosensors in Its Detection: A Comprehensive Review. Anal. Methods.

[59] Matits L., Munk M., Bizjak D.A., Kolassa I.-T., Karrasch S., Vollrath S., Jerg A., Steinacker J.M. (2023). Inflammation and Severity of Depressive Symptoms in Physically Active Individuals after COVID-19—An Exploratory Immunopsychological Study Investigating the Effect of Inflammation on Depressive Symptom Severity. Brain Behav. Immun. Health.

[60] Wang Z., Du Z., Lu R., Zhou Q., Jiang Y., Zhu H. (2024). Causal Relationship between Diabetes and Depression: A Bidirectional Mendelian Randomization Study. J. Affect. Disord..

[61] Xiao X., Wang S., Long G. (2019). C-Reactive Protein Is a Significant Predictor of Improved Survival in Patients with Advanced Non-Small Cell Lung Cancer. Medicine.

[62] Bognár S.A., Teutsch B., Bunduc S., Veres D.S., Szabó B., Fogarasi B., Zahariev O.J., Vörhendi N., Almog O., Hadani Y. (2024). Psychological Intervention Improves Quality of Life in Patients with Early-Stage Cancer: A Systematic Review and Meta-Analysis of Randomized Clinical Trials. Sci. Rep..

[63] Paslaru A.M., Plesea-Condratovici A., Moroianu L.-A., Isailă O.-M., Rebegea L.F., Pavel L.L., Ciubară A. (2025). Mind over Malignancy: A Systematic Review and Meta-Analysis of Psychological Distress, Coping, and Therapeutic Interventions in Oncology. Medicina.

[64] Hungary Today (2024). Internationally Significant Medical Conference in Gyula a Landmark Event. Hungary Today.

[65] Shi-Heng W., Hsu L.-Y., Lin M.-C., Wu C.-S. (2023). Associations between Depression and Cancer Risk among Patients with Diabetes Mellitus: A Population-Based Cohort Study. Cancer Med..

[66] Kern L.M., Edwards A., Kaushal R. (2014). The Patient-Centered Medical Home, Electronic Health Records, and Quality of Care. Ann. Intern. Med..

